# A Rare Occurrence of Relapse Malignant Vascular Mesenchymal Tumors (Rhabdomyosarcoma)

**DOI:** 10.7759/cureus.27525

**Published:** 2022-07-31

**Authors:** Pratiksha K Munjewar, Dr. Ranjana Sharma, Mayur B Wanjari, Vaishnavi V Kantode

**Affiliations:** 1 Medical Surgical Nursing, Srimati Radhikabai Meghe Memorial College of Nursing, Datta Meghe Institute of Medical Sciences, Wardha, IND; 2 Community Health Nursing, Datta Meghe Institute of Medical Sciences (Deemed to be University), Wardha, IND

**Keywords:** deglutition, peripheral nerve sheath tumour, relapsed rhabdomyosarcoma, excision, peripheral nerve, mesenchymal tumour

## Abstract

Relapsed rhabdomyosarcoma (RMS) has several therapeutic challenges. The novel treatment for relapsed RMS was surgical management, chemotherapy, and radiotherapy. Reoccurrence significantly occurs in children and adolescents. RMS occurs anywhere in the body but mostly occurs in the legs, head, neck, urinary, and reproductive systems. Here, we present the case of a 19-year-old female who came to the emergency department with complaints of swelling in the left side of the neck that extended toward the face and left eye, breathlessness, and vomiting for one month. She has a history of peripheral nerve sheath tumor and type 1 diabetes mellitus. Surgical management was done through excision of the mesenchymal tumor surgery, and the patient's prognosis was good.

## Introduction

Rhabdomyosarcoma (RMS) is a mesenchymal malignancy that is correlated with pediatric malignant soft tissue sarcoma of skeletal muscle that arises from the primitive mesenchymal cell [1]. It is a rare cancer that affects children and adolescents; the etiology and risk factors of RMS are still unknown. The prevalence rate of RMS is 0.43 per 100,000 yearly among people under 20 years [2]. There is no standard treatment for RMS, such as tissue biopsy, assessment of post-relapse prognosis, feasibility of the local control measures, and focus on patient preventive care, all of which should be a part of the approach to patient care [3]. Patients treated with multiagent chemotherapy regimens for relapse have the highest chance of long-term cure [4].

## Case presentation

We present a case of a 19-year-old female who came to the emergency department with complaints of swelling on the left side of the neck extending toward the face and left eye, breathlessness, and vomiting for one month. She has the comorbidity of uncontrolled type 1 diabetes mellitus and was taking insulin (Mixtard) daily. She had a history of peripheral nerve sheath tumor in the left posterior triangle of the neck one year back and underwent a diagnostic test, namely, fine needle aspiration cytology (FNAC), which revealed a cytological feature consistent with a benign adnexal tumor. Subsequently, she was operated on for excision of the malignant vascular mesenchymal tumor. Postoperatively, chemotherapy was advised for her, but she refused the therapy due to her personal financial crisis; she was then discharged from the hospital.

**Figure 1:**
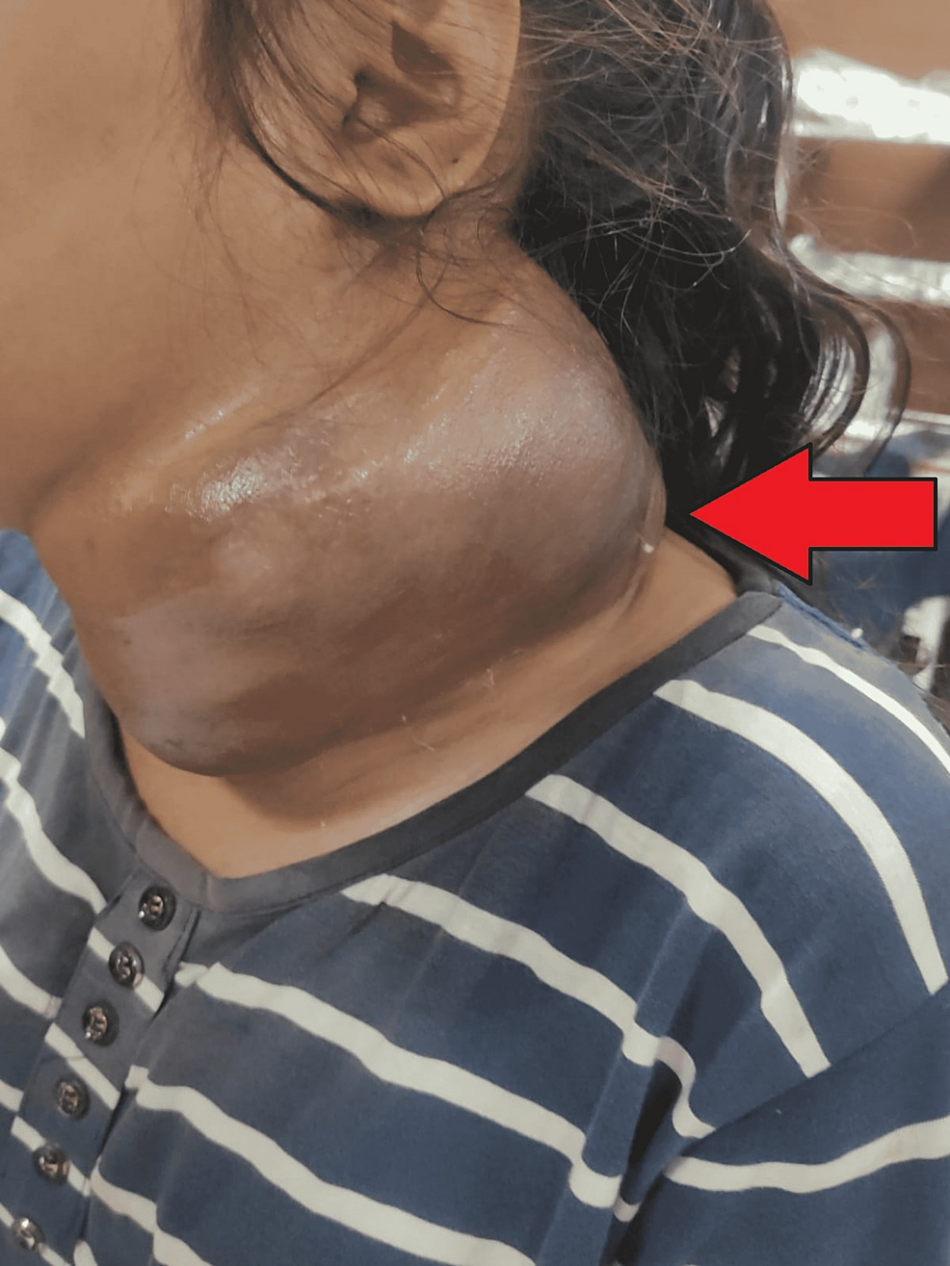
Clinical image showing a cystic swelling of 20 cm x 10 cm on the left side of the neck

After three months of discharge, she revisited with the same complaints, but due to COVID, she was unwilling to be admitted to the hospital. After six months, her swelling started to increase. Contrast-enhanced computerized tomography (CECT) of the neck revealed a large heterogeneously enhancing lobulated ill-defined infiltrative soft tissue mass lesion with an area of necrosis in the region of the left sternocleidomastoid muscle with a highly malignant tumor (Figure 1). After undergoing a thorough investigation, the patient was diagnosed with a malignant vascular mesenchymal tumor (rhabdomyosarcoma), and the physician planned for the tumor excision from the left posterior neck.

On physical examination of the neck, a cystic swelling of 20 cm x 10 cm on the left side of the neck was noted, which was extended over the face involving the area up to the left eye. Shiny skin was present over the swelling; the swelling was non-tender, and there was no discharge from the swelling. The tumor excision from the left posterior neck was done, and after that, she took chemotherapy. Her prognosis was good and was advised for continuous follow-up.

## Discussion

RMS is a malignant soft tissue tumor of the skeletal muscle that was described by Weber in 1854. RMS is most significantly found in the head, neck, genitourinary tract, retroperitoneum, and extremities. Head and neck cancer (HNC) is one of the causes of cancer-related deaths due to conventional therapy resistance [5]. HNC patients were treated with significant surgery, radiation, and chemotherapy. The survival rate for HNC is greater than and less than 50%, depending on the lesion or stage. Nowadays, there is an improvement in the diagnosis and management of HNC, but the survival rate is still improved only marginally over the past year. Therefore, an improvement in the treatment for RMS is essential [6].

RMS begins at mesenchymal and immature cells and develops into skeletal voluntary muscle, which is also known as striated muscle [7]. RMS commonly occurs in children and young adults. The common sites of RMS are the head and neck, urinary and reproductive systems, arms, legs, trunk, etc. [7].

Most commonly, RMS has five types. (1) Embryonal RMS is the most common type, which usually affects the head and neck or reproductive organs. (2) In botryoid RMS, hollow organs are involved (bladder and vagina) [8]. (3) Spindle RMS occurs in the testicles of boys. (4) Alveolar RMS and (5) pleomorphic sarcoma affect arms, legs, and torso, but alveolar RMS is the aggressive type. The pathogenesis of RMS is unknown. It is slightly common in children with genetic disorders like neurofibromatosis or Li-Fraumeni syndrome [8]. Boys are more prone to RMS than girls; sarcomas may occur at a previously treated cancer site. Studies show that risk factors for RMS are high birth rate, exposure to x-ray in the mother’s womb pre-birth, and exposure to certain chemicals in childhood [9].

RMS occurs anywhere in the body, and the symptoms may differ from victim to victim. The symptoms also depend on the size and site of the tumor. The primary symptoms of RMS are a lump or swelling over the affected areas like the neck, chest, leg, back, armpit, or groin area (testicles) [10]. In some cases, RMS may cause pain, redness, and other problems. Tumors surrounding the neck might cause deglutition, dyspnea, difficulty in neck rotation, etc. Protrusion of the eye, blurred vision, discharge from the eye, and redness of the eye are the symptoms of tumor eyesight. Tumor in the bladder or prostate causes symptoms like blood in urine, and cancer in the vagina causes vaginal bleeding. The abdominal tumor causes symptoms like vomiting, gut pain, constipation, etc. [11].

RMS is a highly aggressive malignancy in childhood, which needs early detection. Patients with suggestive symptoms should undergo complete evaluation, including blood tests, FNAC, biopsy, immunocytochemistry test, a genetic test of the tumor, etc. [1]. There are some imaging tests for diagnosing the disease such as ultrasonography, contrast-enhanced CT of the neck, x-ray, MRI, bone scan, positron emission tomography, and color Doppler ultrasound [5].

## Conclusions

In this case, the patient faced a therapeutical challenge for the treatment modalities because of her financial condition. For patients with a first-time relapse, chemotherapy is the most effective treatment. There is a need to do a clinical trial among patients with relapsed RMS to identify the ideal treatment. In this case, the patient was treated with surgical and chemotherapy intervention, which proved to be a novel treatment for the relapse of RMS with a good prognosis. 
